# The Radical Preparation of Cavities

**Published:** 1903-01-15

**Authors:** Harry M. Semans


### THE RADICAL PREPARATION OF CAVITIES.

BY HARRY M. SEMANS, D.D.S.

Read before the Ohio State Dental Society, December 2, 1902.

To-day we hear a battle-cry in the warfare we are waging against our mutual enemy, "The destroyer of teeth, that sounds ominous to the latter's welfare. No doubt every one of us opened our campaign with the determination to do or die; our plans well laid, following the precepts of others, augmented by our own ideas of how the fight should be carried on. But the battle soon changes its aspect; our enthusiasm has passed the ebullition stage, and we settle down to a warfare in which success fluctuates, first to us, then to the enemy. A little skirmish here, heavy fighting there and the ground seems clear of the foe; that part of the field we think is ours. When like thunder out of a clear sky, the fell destroyer dashes on the field, and at it we go again, chagrined somewhat, also our enthusiasm very much dampened, but with dogged determination never-the-less to make good and retrieve our lost vantage. So conquerors again we are, or perhaps we retire after a drawn battle, but woe to us if we retire defeated. Our patients present our field of strife; each individual a skirmish or a battle; disappointing results and unavoidable conditions producing recurrence of decay, etc., bring about our return engagements of warfare.

Other battle-cries have sounded forth, their echoes still reverberating about us. What a stir in the world of dentistry the cry "New Departure" caused with its motto "Truth without fear and without favor." The echoes still faintly sound on the listening ear. But now a new one sounds loud and clear. Hope fills the breasts of many, "No more heart-breaking return battles," they cry, and with expanded lungs and glad hearts they shout the new refrain. Others listen, half doubting; then stumbling, half blind, rush after. Others again receive it with distrust, fearful lest it may carry them into dangerous places. Some say, "It's nothing new, the refrain possibly, that's all."

Thus the new battle-cry, "Extension for prevention," is being received "Radical of the radical," one doubter says, another adds, "I'm not an extremist, I use my best judgment combined with all my skill for each individual case. Radicalism is not my style." Then an extreme advocate of extension for prevention enters the discussion, "If your best judgment is good judgment, and I have no doubt it is, then you are a radical, for radicalism is but the uprooting of all that is false." "No, that's where you err," our doubting friend puts in, "I am conservative to the extent that I do not allow my judgment to be worked by enthusiasm. I am liberal in that I may employ extreme measures if my judgment says so. And you are radical if you bring every case under your extension for prevention theory. Radicalism is my servant, you are its slave."

"But," adds our enthusiastic friend, "It is not in your power to judge as to the possibility of recurrence of decay. If you desire ultimate success you must preclude the possibility of future trouble."

"Oh, well," says Dr. Conservative, "If that is so, let's put it this way: I prepare a simple proximal cavity in an upper central incisor. I cut to the good, clean, strong walls and prepare to fill. But wait, some say, I must cleave away beyond the field of possible future trouble. The tooth is strong now, the general conditions of the mouth are good, but all things are possible in this world, so to the line of safety I go. Again I prepare to fill. But still all things are possible, and the pulp this time is brought within the range of possibility —so out it comes. The third time I prepare to fill. Hold again, sometimes periosteal troubles arise after pulp extermination, how can I tell, better extract and bridge in. And now what direful results we sometimes see and hear about from bridge anchorage. Better remove all the teeth and insert a complete set of artificial teeth. There—I stayed within the range of possibility."

So the discussion goes on, interesting to us, certainly confusing at times, reaching to a point of absurdity if carried as above, all for our ultimate good, no doubt. Like our doubting conservative friend just mentioned, the word "radical" to a great many is a bugaboo. If we are a little uncertain as to what a certain individual is advocating, we call him radical. If we even secretly believe, but fear to openly follow, he is radical. If we think a remedy is needed, but the means heroic—it's radical. If we have arrived at the point of impatience and feel that we must do something final, it is radical mean. To be radical is often held to be less than level-headed, such a one is said to be carried away by enthusiasm regardless of consequences. We often hear and receive advice of this kind: "Always be conservative, don't rush headlong into things." The advice implies: don't be radical.

To-day a number of earnest thinkers in our profession, men who control their earnest thoughts by their long experience in practical results, are advocating what to many seems extreme. Their desire is to do now, the very best that can be done for their patients.

They strive to prevent a recurrence of trouble in the future, instead of merely putting to a stop present trouble. Their desire is more than to ream out and fill a certain particular hole, it is to clear away that particular space so as to prevent the liklihood of having it to do again. They desire to place a filling so that its future will be insured both from its own strength and from the soundness and stability of its neighbors, and to do this they find that something more than good workmanship is necessary, for they have found that good workmanship will often fail. So this constant striving for better results is producing ways and means that seem to many beyond the point of practical every-day necessity.

We find their purpose twofold. First, there is the desire to extend beyond the possible chance of future decay. Second, there is the desire to extend beyond the possibility of a weak filling because of uncertain retention. Many proximal fillings are found to decay at the gingival border and its angles, and occasionally at the incisive end. To many comes the thought, "That's because of poor work," yet there is absolutely no doubt that many good fillings fail there, that is, by good fillings I mean those inserted with good walls. Now we all know that micro-organisms of the decaying food causes caries of the teeth, and we also know that decaying food lodges at the gingival borders of most teeth. A well-inserted filling can by no possible means prevent caries occuring beyond its own field of occupation. V-shaped spaces have been attempted, with little or no results, often bad results. So we are to-day brought face to face with a radical method, which only is this—get beyond the field of possible decay in cavity preparation. Why should we fear the word "radical," it means thoroughness and not reckless enthusiasm as Dr. Conservative contended.

Take a proximal cavity in a superior incisor. A consistent "Extension for prevention" advocate cuts beyond the food-collecting space and passes under the gum festoon, he brings his gingival angles out to the point where decaying food can not lodge. Also the labial and palatial walls may be extended. What is the limit of such preparation? That must be decided by his good judgment. The question is then brought up as to the necessity for such radical treatment, if the field presents no likelihood of future decay. The advocates of extension insist that there is always a chance, even in the very best conditions. Another common field for extension is found at the gingival border of buccal and labial surfaces where erosion plays its fatal game; and where we often find that white crumbling appearance of the enamel. It extends at times clear to the proximal angles and under the gum the entire length. When we find the way to stop the ravages of erosion, we then insure our fillings. It is an every-day occurrence to find bad conditions under many fillings on the proximal surfaces of bicuspids and molars. The number found is measured by extent of practice and time. If the good judgment and skill of the persons who inserted such fillings had been augmented by foresight much trouble would have been averted. It seems to me if we are conscientious in our desire to do what is proper, and yet not resort to extreme treatment in any case, then we must fall back on clairvoyancy, for our ordinary minds can not tell the future.

Take another class of radicalism. Those who advocate extension for prevention, of breaking and splitting away of structure too friable to bear the constant strain; weakened by undercutting and poorly placed retention points. Take a proximal cavity on any of the bicuspids or molars, how often we find those fillings dropping out. The preparation of a step cavity well placed on the occlusal surface puts an end to that trouble. There is no doubt but that all of us dislike to have our professional contemporaries see our unfortunate results and have a chance to shake their heads, talk under their breaths in full view of the patient there with great magnanimity say aloud, "Even the best of us will sometimes have bad results." I will tell you how to get even, treat every case radically, and shake your head over all of their conservative efforts that turn out badly.

Most of us remember the step cavity so well illustrated by Dr. Ruggles before our meeting a few years ago. That was a radical treatment feared by many, and we yet hear men say they resort to it as the last means. It seems to me they should say that they will resort to it if it will cause an avoidance of future trouble. I don't believe any of us to-day, if we have any doubt as to whether a proximal cavity, involving the cutting edge, will remain good, but what will prepare that cavity with a good retaining step, and so it is in many cases, we follow out deep fissures and extend them if we have any doubt as to the future. Two motives then actuate us, one is, that our results may remain good before the eyes of the world; and the other is, that the welfare of the patient should be looked after. The latter however often weighs less than the former. The supposition is that we are all ethical; then why should we hesitate to resort to extreme measures if they are for our own and the patient's good? Is it because of lack of time? Then we slight the work and are not ethical. It surely can not be because of unskillfulness or laziness, for if that is so, we missed our calling. We are then like the farmhand who entered the ministry because he thought he saw the letter "P" in the sky and interpreted it as meaning that he should preach. The presiding elder before very long informed him that instead of preach it ment plow. I wonder if it is because we fear making a bad impression on our patients? There is no doubt at all but that a certain class of patients, and their number is by no means small, are easily impressed with the idea that we are doing them at every turn. The straight-out statement that there is but one possible way, with no side arguments, convinces better than anything else. Many of us dread giving impressions of high prices. I fear we are lowering our standard very much when we allow that to hinder us in our work. We haven't reached that Utopian age yet in which our methods are construed to be for our neighbor's welfare. We are now decidedly living in an age of personal commercialism and our patients are inclined to judge us from their own standard, so if we wish to do our work for the best results, we must impress our patients not only with our skillfulness, but our honesty and integrity. They know after all, but very little ot our ways and means for accomplishing results, but they do soon learn certain characteristics we possess, which sooner or later make or unmake us. They have little or no idea concerning radical preparation of cavities, but they do notice the permanency of fillings. They may fear we are doing more and demanding more than we should, if so, then we are mostly to blame, especially if we have had time to become known to them.

I have not, nor shall I go into detail concerning the methods of preparing a cavity of any kind in a radical way, but will rely upon the models which I have presented to you. The question is simply this: Where should radical preparation be employed, and when?

First then, radical preparation should be done wherever decaying food may find enlodgment, and where very doubtful retention points are in evidence. To the second question: When? I might say that will always depend upon our own individual surroundings and inclination. There is every reason in the world to believe that a filling covering the entire field of possible decay, will last longer and do better than one situated in the middle of that same field, and retention places built into solid tooth structure will insure the stability of a filling better than undercutting in thin or friable walls. When the Spanish bull-fighter takes the bull by the horns does he stab him to the heart, or does he strike him a blow between the eyes and run? Perhaps in the latter case he can reach the fence, and perhaps not. In our dilemma heroic treatment takes our breath away, so we resort to a blow that will hold in check for a while, and trust to our good luck in getting away.

Perhaps some of us are like Dr. Conservative and expect to rely on our good judgment as to the necessity for radical treatment in this often-mentioned field of possible future trouble, even then it is similar to holding the same bull by the horns, we know his temper to be very amiable just now, so we let go and sedately walk to the fence. However no bull ever lived but that he reserved the privilege of showing uncontrolable hatred to all the world whenever he felt like it. Yet we do hate terribly to kill the bull every time.

I acknowledge my illustration to be homely, yet I believe it fits the case very well. Even though we admit the argument yet we dread shaping the conclusion of every case to these arguments, so we solace ourselves by saying, "Radical preparation was not necessary in that case." Perhaps not, but that nice amiable bull may see red some day and those clean, white and strong walls may show caries some day. The pioneer American dentists were radical, the men of forty years ago were radical whenever they departed from the beaten paths of these pioneers. Radicalism is rampant in our profession to-day, and in looking back one hundred years we fail to find a time when it was not in evidence. Radicalism is death to slovenliness, thoughtlessness, carelessness and dishonesty. The dentist who heeds its precepts turns his profession into an art. The man who avoids it altogether does his work as a job-lot.

A conservative is one who desires to maintain existing customs and ways and is opposed to revolutionary or radical means. Radicalism refers to the uprooting of all falsehoods, abuses and wrongfully fixed habits.—*Webster's Dictionary*,

DISCUSSION.

Dr. M. H. Fletcher: If it were possible to discuss the subject of saving the teeth from the results of dental caries from the mechanical point of view, I don't know that I could take any exceptions to the position of the essayist, as technically I would approve his methods so far as he has declared them. It is however the dentists business to not only repair the ravages of decay mechanically but he is in duty bound to do whatever is possible to prevent not only recurrence of decay but its occurrence as well. We all know full well that no mechanical art or skill will avail to save some teeth. It is in these cases where the dentist is forced to exercise his skill as physician and resort to the art of therapeutics or sanitation. Whenever the causes of dental caries are present in the mouth, whether active or not, the physician dentist will resort to such expedients as may be necessary to ward off a threatened attack of illness. It is not final treatment to repair broken down tooth structure by any kind of radical mechanics. For if the active cause of the disease which broke down the tooth tissue in the first instance will very likely prevail over any form of mechanical art however perfect it may be. I assume that in a great many cases failures from radical mechanics would have resulted conservatively if accompanied, or even substituted, by therapeutic or sanitary treatment. It may be also that we are expecting too much from mechanical methods. The physician treats his patient for the present and is not surprised when illness recurs. We should not be greatly troubled when our best technical efforts fail. I do not believe that it is often practicable to produce immunity from dental caries by any operative procedure however skillful. Who for instance believes that recession of the gums can be adequately treated by any kind of radical mechanical treatment. In this case a condition exists which will not be cared for by such a treatment. Mechanical treatment may be utilized in part; but the pathologic conditions must be overcome and their causes obliterated. Physiologic functions must be re-established and the environment must be made and kept healthy. This can not be done by any process of extension for prevention, however radical, it many times needs to be largely supplemented by hygienic treatment of the most scientific kind.

Dr. S. D. Ruggles: Whilst it is true that therapeutic treatment has much to do with conservative practice, dentistry as largely practiced to-day is confined to efforts made to repair the ravages of decay in the teeth, with some it may be merely patch work, and with others most beautiful works of art. It is clearly evident to every one that this effort to repair lost tooth structure should not be inefficiently or carelessly done. I believe if ali tooth tissue which is actually infected, or which is predisposed to infection were carefully removed and the cavity skillfully filled with an indestructable material that the chances for a recurrence of caries would be greatly hindered. Of course there come periodic events in the history of the teeth, as well as accidental causes where no amount of radical mechanics will be entirely adequate. As I understand it, the idea intended to be developed by the author of the theory of radical extension for prevention, is that a well-grounded system of mechanical technique in cavity preparation shall be fundamental and always followed, except in such cases as judgment shall otherwise clearly indicate another form of treatment. This erects a standard of excellence or policy to which doubtful cases may be carried on appeal as it were. In other words it gives us a plan to which we may work and thus it lessens the nervous strain, and insures reasonable results. It makes it possible to lay the foundation for every operator with the completed filling in the minds eye.

Dr. J. B. Beauman: While I believe in the radical preparation of cavities, I have found that most patients want you to "go easy." Extensive cutting of the teeth necessarily produces pain, and usually the more cutting the more intense the pain. It seems to me that a middle ground is tenable. Of course we must take into account the stress that is to be put upon each filling and secure adequate anchorage, we must also eradicate all pathologic tooth structure, and generally such as may be predisposed to decay, at the same time for esthetic and anesthetic purposes we are justified in making limited excavations when judgment approves. I started in the practice of dentistry a medically educated man fifty-five years ago, and I recently saw some fillings that I made at that early date in a hotel with none of the valued facilities we now possess for so-called radical operations, and some of these fillings have saved those teeth all these years. It was not radical work as gaged by present standards. I am not sure but careful and adequate adaptation of gold to the cavity walls is a much more important item than cavity preparation however systematically it may be done. Careful diagnosis and skillful manipulation are both invaluable.

Dr. L. E. Custer: There is one principle which the discussion of the theory of extension for prevention has developed, that to my mind is of the greatest value, and that is the value of a proper enamel margin to support fillings. When we recall the enormous force to which many fillings are or may be subjected the importance of this matter becomes perfectly clear. The work done by Dr. F. B. Noyes on the physical characteristics of enamel is invaluable as a help in forming correct judgment as to when a proper enamel margin has been obtained. The technique of preparation is also much simplified by a correct idea of the structure of the tissues involved. I would commend a study of this feature in all methods of cavity preparation.

Dr. C. B. Hawley: The value of the system of extension for prevention is also brought out in the wonderful improvement it produces in facility for introduction of the filling material. It gives free access, clear view and every opportunity to make perfect adaptation and stability. It is easier to fill against flat walls, than into grooves or acute angles. Retention forms are such as to be readily accessible because not deep or contracted. In proximal cavities of the molars and bicuspids it makes the work easy, and it is here of the greatest value. This method of cavity preparation has also brought non-cohesive foil again into use, as it can be utilized to the best advantage in such preparation.

Dr. J. B. Stephan: This method requires angles and straight walls in the dentin but not necessarily in the enamel. The enamel should in all cases be supported by a layer of dentin beneath, even if it becomes necessary to clean away a considerable surface of the tooth. The dentin may be cut to acute angles, but all surface enamel margins, in outline at least, should be curved and not angular, no acute nor even right angles should even be used, all corners should be curved or rounded.

